# The new surgical robot Hugo™ RAS for total hysterectomy: a pilot study

**DOI:** 10.52054/FVVO.15.4.11

**Published:** 2023-12-13

**Authors:** G Monterossi, L Pedone Anchora, R Oliva, A Fagotti, F Fanfani, B Costantini, A Naldini, D Giannarelli, G Scambia

**Affiliations:** Dipartimento per la salute della Donna e del Bambino e della Salute Pubblica, Fondazione Policlinico Universitario A. Gemelli IRCCS, Rome, Italy; Università Cattolica del Sacro Cuore, Rome, Italy; Facility of Epidemiology and Biostatistics, Fondazione Policlinico Universitario A. Gemelli IRCCS, Rome, Italy

**Keywords:** Robotic surgery, hysterectomy, surgical outcomes, malfunction, safety, Hugo

## Abstract

**Background:**

With the rising popularity of robotic surgery, Hugo™ RAS is one of the newest surgical robotic platforms. Investigating the reliability of this tool is the first step toward validating its use in clinical practice; and presently there arelimited data available regarding this. The literature is constantly enriched with initial experiences, however no study has demonstrated the safety of this platform yet.

**Objectives:**

This study aimed to investigate its reliability during total hysterectomy.

**Materials and Methods:**

A series of 20 consecutive patients scheduled for minimally invasive total hysterectomy with or without salpingo-oophorectomy for benign disease or prophylactic surgery were selected to undergo surgery with Hugo™ RAS. Data regarding any malfunction or breakdown of the robotic system as well as intra- and post-operative complications were prospectively recorded.

**Results:**

Fifteen of the twenty patients (75.0%) underwent surgery for benign uterine diseases, and five (25.0%) underwent prophylactic surgery. Among the entire series, an instrument fault occurred in one case (5.0%). The problem was solved in 4.8 minutes and without complications for the patient. The median total operative time was 127 min (range, 98–255 min). The median estimated blood loss was 50 mL (range:30–125 mL). No intraoperative complications were observed. One patient (5.0%) developed Clavien-Dindo grade 2 post- operative complication.

**Conclusions:**

In this pilot study, Hugo™ RAS showed high reliability, similar to other robotic devices.

**What is new?:**

Present findings suggest that Hugo™ RAS is a viable option for major surgical procedures and deserves further investigation in clinical practice.

## Introduction

In recent years, we have witnessed a progressive increase in gynaecological surgical procedures performed using a minimally invasive approach (Wright et al., 2013). Robotic surgery and the related technical innovations have overcome some of the common limitations of standard laparoscopy thereby extending the remit of minimally invasive surgery even to the most complex cases (Gressel et al., 2020).

It is estimated that in Western Countries, the robotic approach is used in approximately 25% of all hysterectomies performed in hospitals offering robotic surgery (Lim et al., 2020).

The robotic surgery industry is growing rapidly, and various companies have contributed to the technological implementation of new devices (Haig et al., 2020; Fanfani et al., 2015; Hares et al., 2019; Abdel Raheem et al., 2016; Zeng et al., 2021).

In this context, Medtronic introduced one of the newest systems in the market, Hugo™ RAS, which is composed of a system tower, an open console, and four independent arm carts.

There are many possible configurations of this system (Gueli Alletti et al., 2022) making Hugo™ RAS suitable for a wide range of surgical procedures.

Until now, the only data available in the literature concerned a small series of cases regarding urological interventions (including nephrectomy and prostatectomy) (Ragavan et al., 2022) and a report of the first hysterectomy performed with this platform (Monterossi et al., 2022).

Considering this promising initial experience, we decided to set up a pilot study on the reliability and safety of the Hugo™ RAS in gynaecological surgery.

The aim of the present study was to investigate the performance of the new surgical robot, Hugo™ RAS, in a series of total hysterectomies.

## Methods

This was a single-centre prospective study of a series of consecutive total hysterectomies performed at the Division of Gynaecologic Oncology of Fondazione Policlinico Universitario A. Gemelli - IRCCS, Rome, Italy, form March 2022 to June 2022, using the new surgical robot Hugo™ RAS.

All patients suitable for minimally invasive total hysterectomy with salpingo-oophorectomy were considered eligible for the study. Other inclusion criteria were as follows: no absolute contraindications to minimally invasive surgery or Trendelenburg position, American Society of Anesthesiologists (ASA) score not greater than three, and age greater than 18 years.

Patients were excluded from the study if they met at least one of the following criteria: preoperative diagnosis or clinical suspicion of cervical, endometrial, or ovarian cancer.

No specific exclusion criteria were identified in terms of uterine size and previous major abdominal surgery, similar to the criteria commonly applied for the standard laparoscopic approach.

Before the surgical procedures, all patients underwent clinical examination and radiological preoperative workup. Surgical and clinical data were anonymously collected using an electronic database.

IRB approval was obtained, and all patients received a detailed description of the procedure and the risks of robotic surgical intervention, and then gave informed consent, accepting the treatment and authorising anonymisation of the clinical data.

### Surgical Technique

Under general anaesthesia, the patient was placed in the dorsal lithotomy position with both legs supported by Allen stirrups with a Trendelenburg tilt and arms positioned along the body.

The adjustable robotic arms could be individually positioned in different arrangements in space, detached from one another. In our setting, we decided to use three robotic arms, one for the endoscope, and the remaining two for three different instruments: bipolar fenestrated grasper on the left arm, monopolar curved scissors on the right arm, and during the colporrhaphy, a large-needle driver on the right arm after removal of curved scissors.

We used four ports to perform the surgical procedure: first, umbilical access was made thanks to an 11 mm optical port (arm number one); second (arm number two), and third (arm number three) accesses were made with 8 mm titanium trocars in the left and right iliac fossa at a distance of 11cm from the umbilical port. Fourth access was gained with a 5 mm trocar at Palmer’s point, which was used by the table assistant (suction and irrigation, grasping, and closing the uterine artery at the origin with the emoclip). We used a 40° tilt for arms nr.1 and nr.2, coming from the legs of the patient (150° and 220° angles), and +15° of tilt (100° of angle) for arm nr.3, coming from the left arm of the patient.

The first surgeon from the console completely controlled the movement of both the instruments and camera. The first assistant was placed on the patient’s left side. The second assistant placed a uterine manipulator.

**Figure 1:**
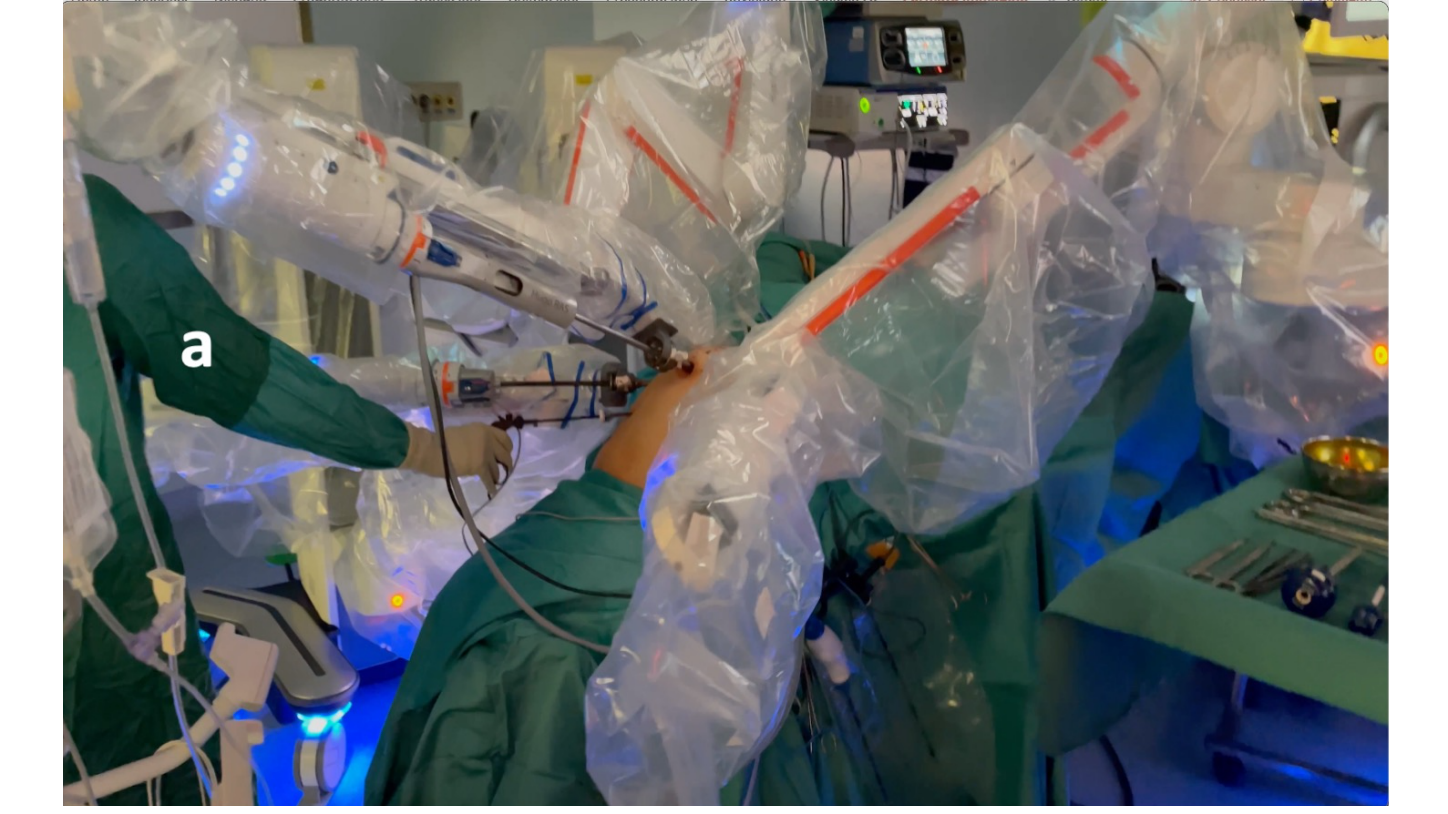
Final system setting. The robotic arms locked to the trocars and the instruments inserted. in this configuration the assistant (a) is positioned to the left of the patient.

**Figure 2:**
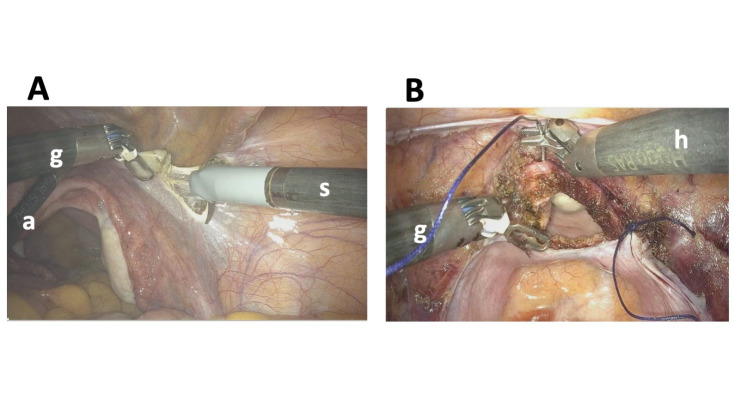
Endoscopic view. A: access to the right retroperitoneum. The assistant (a) moves the uterus to the left, bipolar fenestrated grasper (g) tractions the teres ligament and monopolar scissors (s) incises the broad ligament. B: colporrhaphy. bipolar fenestrated grasper (g) pulls the vaginal angle while the needle holder (h) performs the suture.

In all cases, we adopted a “bridge” port placement (Gueli Alletti et al., 2022) with the “compact” docking configuration where the ancillary port for the bed-side assistant is inserted at Palmer’s point. Figure 1 shows final set-up of the system with the robotic arms locked to the trocars, the instruments inserted, and the assistant positioned to the left of the patient. Figure 2 shows the endoscopic view of the robotic instruments during the opening of the right broad ligament to access the retroperitoneum (A) and during colporrhaphy (B).

Total hysterectomy was performed step-by-step with uterine artery ligation at the origin, according to the previously described technique (Gueli Alletti et al., 2020).

All study procedures were performed by 3 surgeons experienced in both laparoscopic and robotic minimally invasive gynaecological surgery. Before using the Hugo™ RAS, all members of the surgical team (first surgeons, assistants, and nurses) underwent training in the use of this robotic platform specifically organized by Medtronic.

### Data collection

Intra-operative data collection occurred during surgery, and post-operative clinical data were gathered until the patient’s discharge. The patients were then followed up for one month to assess both early and late complications.

Specific time parameters were assessed during the surgical procedure: docking time (DT), defined as the time to change and adapt the robotic setting to the patient, the time to move the robotic arms; and operative time (OT), defined as the interval from the start of the procedure to the suture of surgical incisions, also including DT.

Any malfunctions or breakdowns in the robotic system were documented. Each event was classified into three groups according to the affected system component: software, hardware, and instruments.

In addition, data were recorded regarding the duration of each event, whether it was possible to resolve it, and whether the event required conversion to the standard laparoscopic or open approach.

Any cases of conversion to standard laparoscopy or open approach, also not related to robotic system malfunction, were recorded.

Intraoperative complications were defined as bowel, bladder, ureteric, or vascular injury.

Postoperative pain evaluation during the immediate postoperative period was recorded at 2, 4, 12, and 24 h after surgery using a validated visual analogue pain scale (VAS) and scored from 0 to 10 (0 = no pain; 10 = agonising pain) (McCormack et al., 1988).

The duration of hospital stay was calculated from the day of surgery (day 0) until discharge.

Intra-operative complications were classified according to intraoperative adverse incident classification (EAUiaiC) proposed by the European Association of Urology (Biyani et al., 2020), while post-operative adverse events were classified according to the Clavien-Dindo classification (Clavien et al., 2009).

### Statistical analysis

Descriptive analyses were used to assess the clinical, surgical, and pathological characteristics. Quantitative variables were described using the following measures: minimum, maximum, range, and median. Qualitative variables were summarised using the absolute frequency and percentage of frequency.

It was estimated that eighteen patients were required to detect at least one problem related to malfunction or breakdown of the robotic system, using a 95% confidence level based on a supposed risk of 15% event occurrence (Viechtbauer et al., 2015; Rosner et al., 2011). A sample size of 20 patients was calculated, assuming a study withdrawal rate of 10%. Statistical Package for Social Sciences software (version 25.0; IBM Corporation, Armonk, NY, USA) was used to carry out all statistical calculations.

## Results

From March 9th 2022, a total of twenty women were enrolled in the study and underwent total hysterectomy using Hugo™ RAS. Twelve of the twenty patients (60.0%) underwent surgery due to uterine fibromatosis, 3 (15.0%) due to endometrial hyperplasia, and 5 (25.0%) underwent prophylactic surgery due to BRCA-1 mutation.

As shown in Table I, the median age was 51 years, and the median BMI was 24 kg/m².

**Table I:** Clinical and pre-operative characteristics of the series.

	N. (%)
All cases	20
Indication to surgery	
- uterine fibromatosis	12 (60.0)
- endometrial hyperplasia	3 (15.0)
- BRCA mutation	5 (25.0)
**Median Age**, years (range)	51 (43 – 62)
**Median BMI**, kg/m² (range)	24 (19 – 32)
Previous abdominal surgery	
- Caesarean section	3 (15.0)
- Open appendectomy	3 (15.0)
- LPS appendectomy	4 (20.0)
- LPS unilateral salpingo-oophorectomy	1 (5.0)
- No previous surgery	9 (45.0)
ASA score	
- 1	1 (5.0)
- 2	19 (95.0)
Median uterine length, mm (range)	77.5 (62.9– 125)

Eleven patients (55.0%) had previously undergone an abdominal surgery. The vast majority of patients (19, 95.0%) had an ASA score of two, and only one (5.0%) had an ASA score of one.

The median uterine length on preoperative ultrasound was 77.5 mm (range: 62.9 mm – 125 mm).

Most patients (14, 70.0%) underwent concomitant bilateral salpingo-oophorectomy, while 5 (25.0%) underwent bilateral salpingectomy. One patient (1, 5.0%) who had previously undergone unilateral salpingo-oophorectomy at childbearing age underwent concomitant unilateral salpingo- oophorectomy.

Among the entire series, a fault occurred in monopolar scissors in one case (5.0%, 95% confidence interval: 0-14.5%) (Table II).

**Table II:** Robotic system malfunctions.

	System’s component		
	Software	Hardware	Instruments
All events	0	0	1
Time required to solve malfunction (minutes)	/	/	4.8
Cases requiring conversion	/	/	0

After its placement, the instrument was not recognized by the system; therefore, the surgeon was not able to move and control it. The instrument was placed in the contralateral robotic arm, which resulted in the same error. Finally, the scissors were changed to a new scissor to solve the error. The problem took 4.8 minutes to solve without complications for the patient and without any need for surgical conversion.

Median DT was 7.5 minutes (range:3.5 minutes – 10 minutes). The median total OT was 127 min (range:98 – 255 min) (Table III).

**Table III:** Intra- and post- operative surgical outcomes.

	N. (%)
Median docking time, minutes (range)	7.5 (3.5 – 10.0)
Median operative time, minutes (range)	127 (98 – 255)
Median estimated blood loss, mL (range)	50 (30 – 125)
Intra-operative complications	
- grade 0 (no IAE)	20 (100)
- grade 1	0 (0.0)
- grade 2	0 (0.0)
- grade 3	0 (0.0)
- grade 4	0 (0.0)
- grade 5	0 (0.0)
Conversion	
- LPS	0 (0.0)
- Open	0 (0.0)
Median pain score*, (range)	
-2h	2 (1 – 3)
-4h	2 (1 – 3)
- 12 h	4 (1 – 8)
- 24 h	2 (1 – 5)
Time to discharge, days (range)	2 (2 – 3)
Post-operative complications	
- grade 1	1 (5.0)
- grade 2	0
- grade 4	0
- grade 5	0

IAE: intraoperative adverse event; *according to visual analogue pain scale (VAS).

The median estimated blood loss was 50 mL. No intraoperative complications were recorded.

Pain decreased after surgery, with 2-, 4- and 12- hours median VAS scores of 2, 2, and 4, respectively. At 24 h, the median VAS score was 2 (range:1 – 5).

Most patients were discharged on the second postoperative day, resulting in a median time to discharge of 2 days (range: 2 – 3 days).

During the follow-up period, one patient (5.0%) experienced a Clavien-Dindo grade 2 late complication. A urinary infection appeared on the seventh postoperative day and was treated with a single oral administration of 3g Fosfomycin.

## Discussion

Following the Hippocratic injunction “primum non nocere,” when a new technology is available in the field of medicine, the first goal is to test its safety.

Owing to their advantages, robotic technologies are emerging in many fields of medicine (Ning et al., 2021; Takebayashi et al., 2022; Gassert et al., 2018) but, currently, these systems are used mainly in surgery. Robotic surgery is based on sophisticated engineering, which is significantly more complex than traditional laparoscopic instruments in terms of both hardware and software. Therefore, a robotic system may have a higher probability of experiencing dysfunction (Buchs et al., 2014).

This is the first study exploring the use of the Hugo™ RAS in gynaecology, and, for this reason, the study was designed setting as the main outcome all possible adverse events linked to the breakdown of the various components of the platform.

The results of this first experience were encouraging, showing that the Hugo™ RAS is safe and reliable in the field of gynaecological surgery. Only a single negligible instrument error was recorded and it was resolved in a few minutes, without any adverse impact to the patient.

The literature reports that malfunctions of the Da Vinci® robotic system, currently the most used robotic surgery platform worldwide, occur in 2.4% – 4.5% of cases (Buchs et al., 2014; Agcaoglu et al., 2012; Kim et al., 2009; Chen et al., 2012). Data from a study conducted on more than 500 general surgical procedures performed using the Da Vinci® system showed that 50% of malfunctions were related to endoscopic instruments (Buchs et al., 2014). Fortunately, this kind of breakdown was resolved in all cases with instrument replacement without consequences for the patients.

However, it must also be acknowledged that equipment failure is a common event in laparoscopy during the everyday clinical practice (Paracchini et al., 2021) requiring the replacement of the just the affected instruments.

In the present series, the Hugo RAS system seemed reliable in terms of surgical outcomes. No intraoperative complications were recorded, and the only post-operative complication was urinary tract infection, probably resulting from Foley catheter placement.

Intraoperative blood loss and OT were also comparable to the mean reported in cases of hysterectomies performed using the Da Vinci® system or standard laparoscopy (Albright et al., 2016).

In this initial experience with the Hugo™ RAS, the mean DT was less than 10 min. Similar time to complete docking was reported for other robotic systems composed of independent arms (Fanfani et al., 2016).

The Post-operative recovery outcomes were also satisfactory. As a matter of fact, also thanks to a low level of post-operative pain, most patients were discharged on the second day after surgery. It should be emphasised that most of the patients could have been discharged on the first post-operative day. However, in our country, hysterectomies are reimbursed by the National Health System only if patients have a hospital stay of two days or more after surgery.

Since the beginning of the adoption of the Hugo™ RAS in our institution, we established the present study with the main objective of assessing the reliability of the robotic system, after which we enrolled the first 20 cases. This approach to a new platform could be a solid initial step in future research on this device.

All patients enrolled in the study underwent total hysterectomy. We chose to test this new system for this type of surgery because it is the most common gynaecological surgical procedure (Cohen et al., 2014). Moreover, total hysterectomy is a complex surgery composed of surgical space preparation, coagulation and cutting of vessels and ligaments, and excisional and reconstructive steps that allow testing of various surgical performances of the Hugo™ RAS.

The sample size was calculated to identify at least one breakdown of the system, similar to other studies investigating new devices (Fiorentino et al., 2006; Unal et al., 2022; Gueli Alletti et al., 2018). The identification of one malfunction supports the accuracy of the study and the weight of its findings.

We know that the present study represents an initial and exploratory experience of the new robotic system, just as we know that further cases are needed in order to establish the safety and reliability of this new surgical instrument.

Further studies should investigate the use of the Hugo™ RAS in other fields of gynaecological surgery, such as urogynaecology, endometriosis, and gynaecological oncology.

Certainly, it is too early to draw definitive conclusions. However, gynaecological surgery using the Hugo™ RAS seems to be safe and effective. A larger case series would confirm the present data and determine whether this technology offers additional benefits.
